# Chronic pulmonary aspergillosis in tea population of Assam

**DOI:** 10.1371/journal.pntd.0012756

**Published:** 2025-01-08

**Authors:** Aishwarya Selvasekhar, Reema Nath, Gourangie Gogoi, Pronami Borah

**Affiliations:** 1 Department of Microbiology, Assam Medical College & Hospital, Dibrugarh, Assam, India; 2 Department of Community Medicine, Assam Medical College & Hospital, Dibrugarh, Assam, India; 3 Department of Radiology, Assam Medical College & Hospital, Dibrugarh, Assam, India; Universidad de Antioquia, COLOMBIA

## Abstract

**Background:**

Chronic pulmonary aspergillosis (CPA) is a disease commonly caused by *Aspergillus fumigatus* and other *Aspergillus* species characterized by cavitary lung lesions. Tea garden population is an agrarian population of Assam, mostly associated with tea plantations. Assam is a major tea-producing state with 803 tea gardens producing approximately 50% of the total tea in India, of which 177 are present in the Dibrugarh district alone. Tuberculosis is common in tea garden workers. This community-based cross-sectional study in the tea garden community of Dibrugarh was done to find the prevalence of *Aspergillus* IgG antibodies and CPA cases in individuals with chronic respiratory symptoms.

**Methodology and principal findings:**

Patients visiting 3 tea garden hospitals and 2 referral hospitals with chronic cough and/or haemoptysis, weight loss/fatigue, and other respiratory symptoms for a duration of 3 months or more were included in this one-year study. Serum samples were tested by Immunocap Phadia 200 for *Aspergillus fumigatus*-specific IgG antibodies. CPA cases were diagnosed based on clinical, radiological, and serological criteria. Out of 128 samples, seropositivity was seen in 41 (32.0%) patients (cutoff value: 27 mgA/l). Male preponderance (1.6:1) with a mean age of 41.9 (±15.69) was observed. Haemoptysis and fatigue were significant symptoms seen (p-values: 0.0086 and 0.0098, respectively). *Aspergillus fumigatus*-specific IgG antibody was found to be significantly high in 29 out of 76 patients (38.1%) with a history of tuberculosis. Amongst them, seropositivity with active TB was 5 out of 27 patients (18.5%), and seropositivity with post-TB was 24 out of 49 patients (48.9%). Chronic cavitary pulmonary aspergillosis was the predominant type (38.1%). Proven CPA (clinically, radiologically, and serologically positive) were 22 (17.1%, 95% CI 10.7%–26.0%), and possible CPA (clinically and serologically positive but without radiological data) were 19 (14.8%, 95% CI 8.9%–23.1%).

**Conclusion:**

A high prevalence of CPA (60 per 100 000) was detected. High *Aspergillus* seropositivity of 48.9% was seen in the post-TB population. *Aspergillus*-specific IgG antibody testing is the only confirmatory method for diagnosing CPA, which is available in limited centres in India. *Aspergillus* seropositivity should be detected in post-TB patients presenting with chronic respiratory symptoms.

## Author summary

Chronic pulmonary aspergillosis is a chronic lung disease that occurs commonly in pre-existing lung cavities. It mostly occurs in post- or active-tuberculosis patients and shares similar clinical features with tuberculosis. Many tuberculosis cases are treated without microbiological evidence; such cases might be CPA cases that are misdiagnosed as tuberculosis. CPA-related mortality and morbidity can be reduced by detection of *Aspergillus*-specific IgG antibody. The test for detection of antibodies is still not available in resource-limited health setups. The disease is underdiagnosed because of the unavailability of this test. This study evaluated the prevalence of *Aspergillus* IgG antibody and CPA in clinically significant patients of tea garden community of Assam for the first time and found it to be 32.03% and 17.18%, respectively. *Aspergillus fumigatus* seropositivity was found to be 48.9% in post-tuberculosis patients in our study. Haemoptysis and fatigue/tiredness were significant symptoms associated with CPA in our study.

## Background

*Aspergillus fumigatus* is responsible for several pulmonary diseases that depend on the immune status of the patient. Chronic pulmonary aspergillosis is a condition due to this fungus that occurs in apparently immunocompetent patients, whereas invasive pulmonary aspergillosis usually occurs in immunocompromised patients. Localised immune status alteration is notable in pulmonary tuberculosis patients in the form of residual cavities that support the growth of the fungi. Other risk factors are known, which include chronic obstructive pulmonary disease (COPD), asthma, sarcoidosis, etc.

*Aspergillus fumigatus* is the causative agent of CPA. However, culture of sputum from CPA patients often does not grow the fungi. Hence, antibody detection for the same combined with radiological features suggestive of fungal infection in chest radiographs and/or tomography became the confirmatory criteria for CPA diagnosis.

Though various studies are done worldwide in advanced centres to detect chronic pulmonary aspergillosis (CPA) by *Aspergillus*-specific IgG antibody testing, studies from resource-limited settings are very few. Therefore, the cases are underdiagnosed or diagnosed late only when massive haemoptysis occurs or when the patient is in severe respiratory distress.

### Hypothesis

Tea garden population is an agrarian population of Assam, mostly associated with tea plantation. Assam is a state in the north eastern part of India which is a major tea producer state. A total of 803 tea gardens are present in Assam out of which 177 are present in the Dibrugarh district alone. State of Assam accounts for more than 50% of the tea production of India. Dibrugarh is famously known as “Tea City of India”, and is surrounded by the tea estates. It also has a high burden of tuberculosis especially in the people working in tea gardens (Nikshay portal, Govt of India).

Hence, this cross-sectional study was performed from the tea garden community of Dibrugarh district with the following aim and objective:

1. Detection of *Aspergillus fumigatus* IgG antibody in patients with chronic respiratory symptoms.
2. Detection of Chronic pulmonary aspergillosis in this population.

## Methodology

### Ethics statement

Ethics approval was obtained from the Institutional Ethics Committee (Human) with registration number ECR/2003/Inst/AS/2024 issued under New Drugs and Clinical Trial Rules, New Delhi, India, 2019. Approval to conduct this study by IHEC was granted on 16^th^ February 2023. Informed consents (in the form of signatures or thumb prints) were obtained from each and every individual in their own language (Assamese).

### Study design

This was a community-based cross-sectional study that included four tea gardens and associated tea garden hospitals (3 tea garden hospitals and 2 referral hospitals) situated within a 15 km radius from a teaching hospital and a tertiary care centre, Assam Medical College and Hospital, where this study was conducted.

### Study setting

Each tea garden hospital was visited for sample and data collection until the required sample size was fulfilled. Every subsequent patient with significant chronic respiratory symptoms of more than 3 months duration was taken up for the study, and house-to-house sample collection was done as and when required. Radiological test data were collected. Patients in whom chest X-ray/HRCT was unable to be performed were also included in the study. Before initiating the study, a meeting was arranged with the in-charge doctors, nursing staff, Accredited Social Health Activist (ASHA) workers, and other staff of the tea garden hospitals. The details, such as the purpose of the study, samples that will be required, and the type of patients who will be included, were discussed in the meeting in lay terms in their local language.

### Sample size

The sample size was calculated to be 138 using Fisher’s formula (“n = Z^2^ 4pq ÷ d^2^”) with a prevalence of 10% taken from the latest Indian study [1]. The coefficient interval was 95% with a margin of error of 0.05. A total of 128 samples were collected during the study period. Adults with chronic respiratory symptoms as mentioned in the above specific criteria were included.

### Case definition

Chronic pulmonary aspergillosis can be diagnosed on the basis of four required categories as given by the ‘Global Action Fund for Fungal Infections’ (GAFFI) and used by the CDC (Centre for Disease Control) as follows [2]:

1. Clinically, major required symptoms are persistent **cough** with or without **haemoptysis** and **weight loss** for a minimum period of 3 months. Others commonly present are fatigue/tiredness, productive sputum, dyspnoea, and chest pain (but not a required criteria).
2. Microbiologically, seropositivity of *Aspergillus*-specific IgG antibody and/or microscopy of sputum samples showing (dichotomous branching and septate hyphae) hyphae of *Aspergillus* and/or growth of *Aspergillus* species in more than 2 sputum samples or other respiratory samples such as bronchoalveolar lavage (BAL), lung biopsy, and fluid aspirated from cavity (if present) or from pleural effusion is required.
3. Radiologically, chest imaging that shows either cavitation or a fungal ball inside the cavity or thickening of the pleura or fibrosis or infiltrates around the cavities.
4. A negative sputum smear, GeneXpert, and/or mycobacterial culture in order to rule out mycobacterial infection is necessary.

**Proven CPA:** Patients with chronic respiratory symptoms of cough and/or haemoptysis, weight loss, and/or other respiratory symptoms with or without underlying significant respiratory conditions, *Aspergillus* IgG seropositivity (>27 mgA/l in Immunocap testing), and radiological evidence from HRCT/chest X-ray were taken as ‘Proven CPA cases’ in our study.

**Possible CPA:** Cases with risk factors and significant respiratory symptoms of cough and/or haemoptysis, weight loss, and/or other respiratory symptoms with *Aspergillus fumigatus*-specific IgG seropositivity where radiological test records were not found were included as ‘Possible CPA’ in our study.

**Fig 1:**
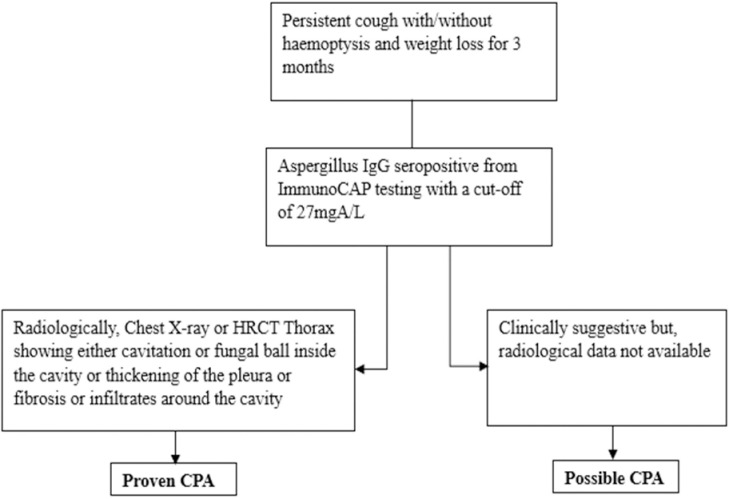
Diagnostic chart for detection of proven and possible CPA cases.

The following figure (Fig 1) depicts the diagnostic guidelines followed for the detection of proven and possible CPA cases in this study.

For concurrent CPA and pulmonary tuberculosis infections, *Aspergillus* seropositivity, chest X-ray/Computed Tomography (CT) scan showing any of the defining features of CPA as described by GAFFI, and mycobacterial positivity (a positive sputum smear, GeneXpert) were taken in our study.

CPA can either present as a co-infection with TB or as a complication after anti-TB therapy [3]. Hence, the following periods of estimation can be considered while evaluating cases of CPA as proposed by Denning and Ray in their study [4]:

- Early (2–6 months during ATT).
- Late (7–12 months after starting ATT).
- 2–5 years after completion of ATT.
- More than 5 years after diagnosis of TB.

In our study, we have labelled the patients belonging to the early period as “CPA with active TB” and those belonging to the rest of the periods as “CPA with post TB.”

#### Inclusion criteria.

1. Patients with respiratory symptoms like weight loss, fatigue/tiredness, cough, haemoptysis, and breathlessness for a period of 3 months.
2. Clinically suspected to have lower respiratory tract infection or treated TB with persistent respiratory symptoms.

#### Exclusion criteria.

Infants and children below 18 years.

### Data collection tools

#### Proforma.

With the help of a preformed Proforma, the socio-demographic data, significant tuberculosis history, and history of other premorbid conditions were collected (Enclosed as S1 Annexure).

#### Consent.

Before proceeding with any kind of tests, informed consent was obtained from the subjects (Enclosed in S2 Annexure).

### Laboratory diagnosis

#### Processing of samples.

Collected serum samples were stored at −20°C. Estimation of *Aspergillus fumigatus*-specific IgG antibody was done by Immunocap PHADIA 200 (Thermo Fisher, Uppsala, Sweden) according to the manufacturer's instructions in Advanced Mycological Diagnostic and Research Centre (AMDRC) serology lab, Assam Medical College, Dibrugarh.

#### Data analysis.

The data were manually collected with the help of proforma and were pooled in Microsoft Excel sheets, coded, tabulated, and analyzed with the help of Epi Info Version 7.2.4.0 software. The numerical variables were summarized by mean and standard deviation, while categorical variables were summarized by frequencies and proportions. Socio-demographic data, co-morbidities, clinical features, and radiological data were compared between seropositives and seronegatives for *Aspergillus* IgG antibody using Chi-square or Fisher’s exact test and odds ratio as and when required. Univariate analysis was done on the various variables of the study and on the radiological features observed and has been displayed in the tables and figures below.

## Results and discussions

Male patients 80 (62.5%, 95% CI 63.4%−99.6%) outnumbered female patients 48 (36.8%, 95% CI 35.4% to 63.6%) by a ratio of 1.6:1.

**Fig 2:**
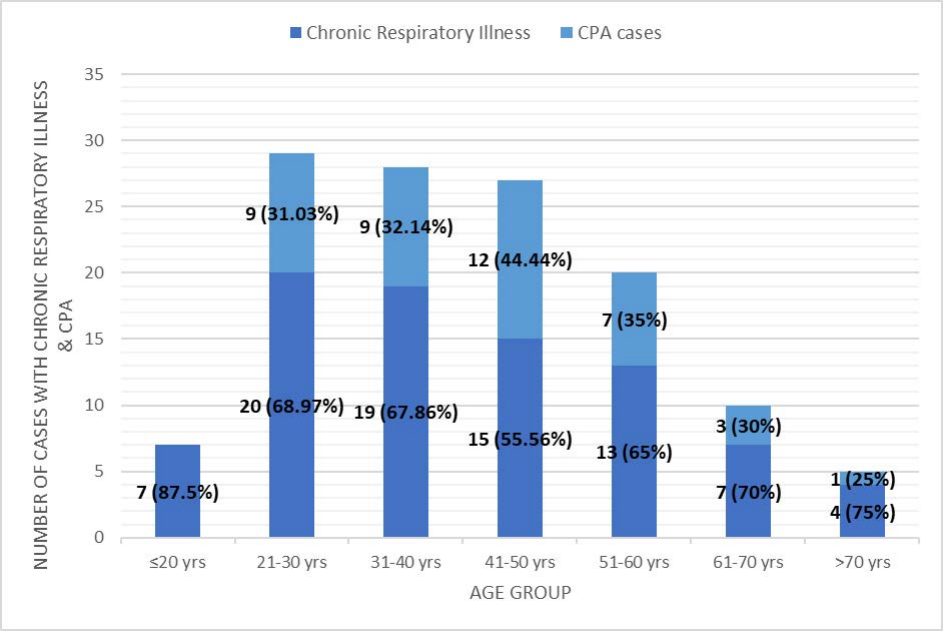
Age‐wise distribution of the patients with chronic respiratory symptoms and CPA cases.

**Fig 3:**
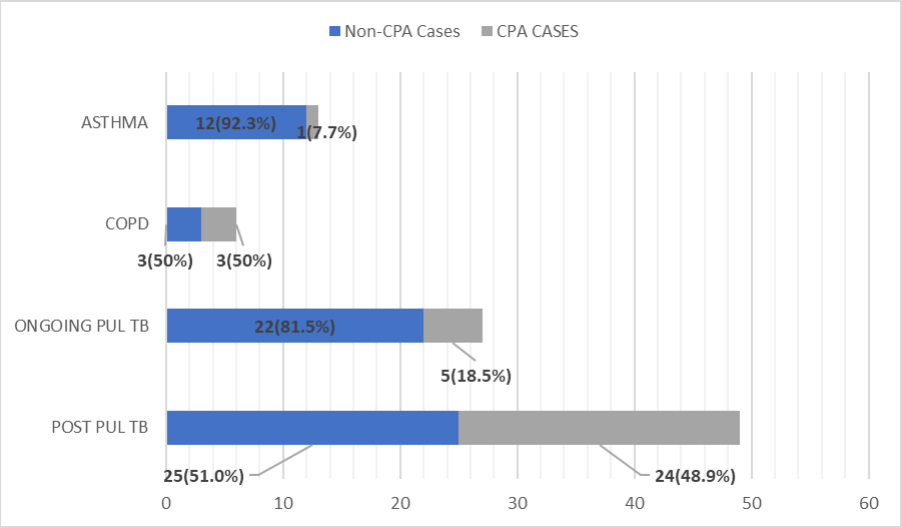
Patients with risk factors of respiratory illness in relation to *Aspergillus* IgG status.

**Fig 4:**
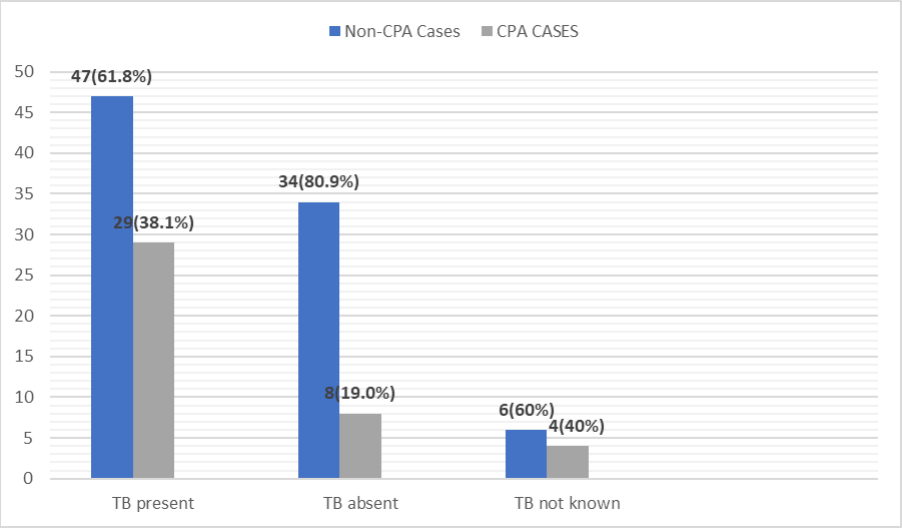
CPA Patients with a history of TB.

**Fig 5:**
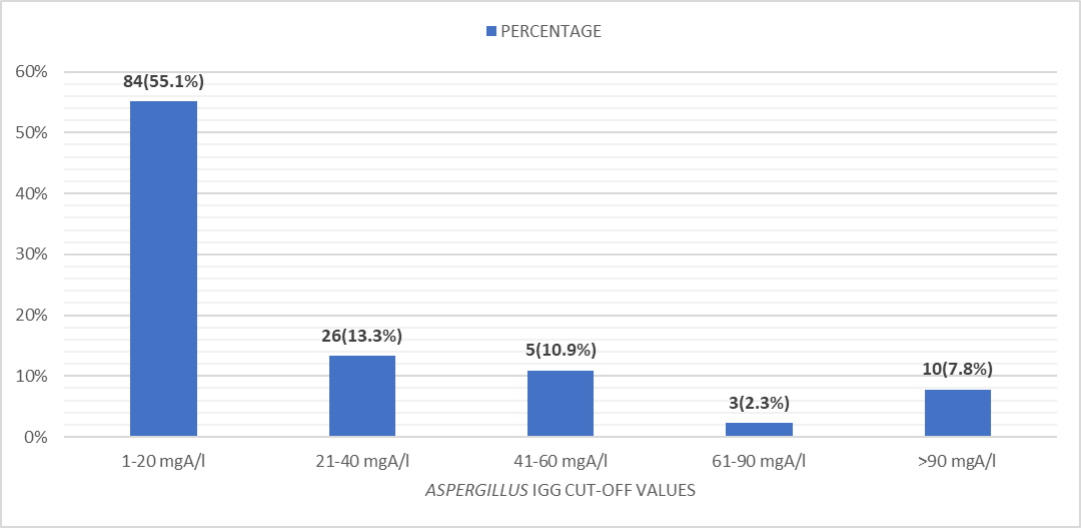
Range of *Aspergillus* IgG antibody in the study population (n = 128).

**Fig 6:**
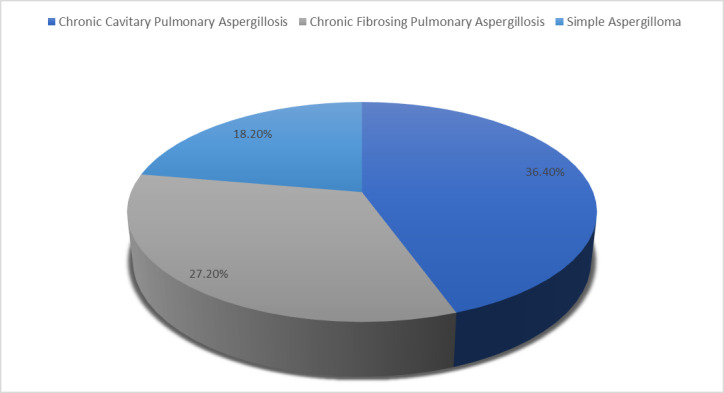
Distribution of proven CPA cases based on their types.

The results and observations of this study are tabulated in Tables 1–4 and/or shown as Figs 2–6 below:

**Table 1:** Population studied from various tea gardens/tea garden hospitals.

Name of the Tea gardens/Garden hospital	Patients studied (%)
Greenwood/Greenwood Garden hospital	19 (14.8%)
Bokel/Bokel garden hospital	18 (14.1%)
Maijan/Maijan garden hospital	9 (7.0%)
Chabua/Referral Hospital and Research Centre (RHRC)	20 (15.6%)
Patients from other tea gardens presenting to RHRC	30 (23.4%)
Referred to Assam Medical College and Hospital from various tea gardens	32 (25%)
Total	128 (100%)

**Table 2:** Distribution of significant clinical features among the patients with chronic respiratory symptoms and seropositives.

Clinical features	Total patients (%)	*Aspergillus* IgG positives n = 41(%)	p-value
Weight loss	30 (23.4%)	8 (19.5%)	0.4729
Fever	43 (33.6%)	12 (29.3%)	0.4776
Dyspnoea	51 (39.8%)	15 (36.6%)	0.6055
Chronic cough	85 (66.4%)	30 (73.2%)	0.2679
Haemoptysis	27 (21.1%)	15 (36.6%)	**0.0086**
Fatigue/tiredness	56 (43.7%)	25 (61%)	**0.0098**
Others (Chest pain)	13 (10.4%)	3 (7.3%)	0.4691

**Table 3:** Univariate analysis of radiological findings of the study.

Variables (radiological findings)		*Aspergillus* IgG			Odds ratio (95%CI)	p-value
		Total	Positives	Negatives		
Cavity formation (single & multiple)	Present	14	12	2	9.00 (1.64 to 49.13)	**0.0112**
	Absent	25	10	15		
Fungal ball	Present	11	11	0	30.36 (1.62 to 565.90)	**0.0222**
	Absent	28	12	16		
Soft tissue mass	Present	3	3	0	5.63 (0.27 to 116.99)	0.2639
	Absent	36	20	16		
Consolidation	Present	6	5	1	4.70 (0.49 to 44.78)	0.1779
	Absent	33	17	16		
Bronchiectasis	Present	10	8	2	4.28 (0.77 to 23.74)	0.0957
	Absent	29	14	15		
Fibrotic opacities	Present	10	7	3	2.17 (0.46 to 10.11)	0.3207
	Absent	29	15	14		
Pleural thickening	Present	5	4	1	3.15 (0.31 to 31.29)	0.3258
	Absent	34	19	15		
Pleural Effusion	Present	9	3	6	0.28 (0.06 to 1.39)	0.1223
	Absent	30	19	11		

Few HRCT chest images of the patients have been included as S1–S6 Figs in supplementary files.

**Table 4:** Univariate analysis of demographic variables, comorbidities, risk factors and clinical features of the study subjects.

Variables		*Aspergillus* IgG			Odds ratio (95%CI)	p-value
		Total	Positives	Negatives		
**Gender**	Male	80	29	51	1.70 (0.76 to 3.78)	0.1888
	Female	48	12	36		
**Age**	<50 years	94	30	64	0.98 (0.42 to 2.26)	0.9626
	>50 years	34	11	23		
**Marital status**	Married	107	37	70	2.24 (0.70 to 7.16)	0.1714
	Single	21	4	17		
**Occupation**	Tea garden/Tea factory	60	16	44	0.62 (0.29 to 1.33)	0.2233
	Others	68	25	43		
**Smoking**	Present/Occasional	33	13	20	1.55 (0.68 to 3.55)	0.2945
	Absent	95	28	67		
**Alcohol**	Present/Occasional	43	12	31	0.74 (0.33 to 1.66)	0.4776
	Absent	85	29	56		
**HIV status**	Present	3	2	1	4.00 (0.33 to 47.29)	0.2713
	Absent	51	17	34		
**Total TB (ongoing and post)**	Present	76	29	47	2.62 (1.06 to 6.44)	**0.0355**
	Absent	42	8	34		
**Post TB**	Present	49	24	25	2.93 (1.34 to 6.42)	**0.0070**
	Absent	69	17	52		
**Asthma**	Present	13	1	12	0.22 (0.02 to 1.83)	0.1636
	Absent	70	19	51		
**COPD**	Present	6	3	3	2.68 (0.49 to 14.47)	0.2507
	Absent	70	19	51		
**Fever**	Present	43	12	31	0.74 (0.33 to 1.66)	0.4776
	Absent	85	29	56		
**Weight Loss**	Present	30	8	22	0.71 (0.28 to 1.78)	0.4729
	Absent	98	33	65		
**Dyspnoea**	Present	51	15	36	0.81 (0.38 to 1.75)	0.6055
	Absent	77	26	51		
**Chronic cough**	Present	85	30	55	1.58 (0.70 to 3.59)	0.2679
	Absent	43	11	32		
**Hemoptysis**	Present	27	15	12	3.26 (1.35 to 7.90)	**0.0086**
	Absent	94	26	68		
**Fatigue/Tiredness**	Present	56	25	32	2.73 (1.27 to 5.86)	**0.0098**
	Absent	72	16	56		
**Chest pain**	Present	13	3	10	0.60 (0.15 to 2.33)	0.4691
	Absent	115	38	77		

Images of ImmunoCAP reports showing calibrator curves and *Aspergillus* IgG levels of a few patients have been included as S7 and S8 Figs in supplementary files.

## Discussion

### Prevalence of chronic pulmonary aspergillosis

Twenty-two out of 41 (53.6%) serologically and clinically significant patients could be followed up with HRCT/chest-X ray and all of them (100%) had radiological as well as clinical features consistent with CPA. As this study was done in a resource- and time-limited setting of 1 year, the radiology follow-up was limited. The rest of the 19 (46.3%) serologically significant cases with clinically significant respiratory symptoms without radiological data were categorized as possible CPA [2]. The total population of the four tea gardens (namely Greenwood, Maijan, Bokel and Chabua tea gardens) included in the study was 20,903; where 14 cases of CPA were detected (out of 66 clinically suspected patients from the four tea gardens), which gives us a prevalence of 60 per 100 000, which is higher than the global prevalence reported. The remaining 62 clinically suspected patients of the tea population were from two referral hospitals, from whom 27 CPA cases were detected. Global prevalence of CPA is reported to be 42 per 100 000 [5], while in India it was estimated at 0.02% in 2011 by Denning et al [6]. Revised estimation of 5-year prevalence of CPA in India related to TB was reported as 1.5 million cases [7]. In the US and Europe, the prevalence is <1 per 100 000 (0.6) and 42.9 per 100 000 in the African nations of the Democratic Republic of Congo and Nigeria [5]. In another study done in France by Maitre T et al., the prevalence was estimated to be 5.02 per 100 000 in 2018 [8].

A total of 41 out of 128 patients (32.0%) were categorized as CPA (22 Proven CPA 17.1%, 95% CI 10.7% −26.0% and 19 Possible CPA 14.8%, 95% CI 8.9%−23.1%) out of these patients. In our study, the prevalence of *Aspergillus* IgG seropositivity in post-TB population with chronic respiratory symptoms attending garden hospitals is 48.9% (24 out of 49 post-TB patients). One systematic meta-analytic review from Asia has reported the prevalence of *Aspergillus* antibodies in post-TB patients as 14.7% [9]. A study from North India by Sehgal et al. showed a prevalence of CPA as 10.3%, and another from New Delhi by Jha et al. showed a prevalence of 21.5% in post-tuberculosis patients [1,10]. A recently published review, which gives the revised burden from India, determines the rate of developing CPA after pulmonary TB diagnosis to be 10% in the first year and 1.5% in the subsequent 2–5 years [4]. Other studies from Sierra Leone, Kenya, and Ghana showed prevalence in the tuberculosis population of 20.8%, 19.7%, and 50%, respectively [11–13].

The tea garden community of Assam is an agrarian community, the adult population of which works closely in tea plantations. Many dependents of this population also work outside the tea gardens, engaged in various supportive occupations for the society, like masons, plumbers, carpenters, manual labourers, etc. This study is done for the first time in the tea population of Eastern Assam to get an insight into the role of *Aspergillus* species, commonly *Aspergillus fumigatus,* in the causation of respiratory illness. The *Aspergillus fumigatus*-specific IgG antibody level is helpful in diagnosing several respiratory disorders, mainly CPA (chronic pulmonary aspergillosis), ABPA (allergic bronchopulmonary aspergillosis), chronic *Aspergillus* sinusitis/fungal ball, hypersensitivity pneumonitis/extrinsic allergic (broncho) alveolitis, and *Aspergillus* bronchitis (in cystic fibrosis and bronchiectasis) [2]. Out of 128 patients, 44 (34.4%) patients had antibody levels of more than 21 mgA/l while 41 (32.0%) patients had levels more than 27 mgA/l. In a recent study in Taiwan, the cutoff value of significant antibody was determined to be as low as 20 mgA/L [14]. In a couple of latest Indian studies from North India by Agarwal et al. and Sehgal et al., it was determined as 27 mgA/l for diagnosis of CPA [15,16].

In our study, *Aspergillus fumigatus*-specific IgG antibody was found to be significantly high (>27 mgA/l) in 29 out of 76 patients (38.1%) with a history of tuberculosis (p-value: 0.0355). Amongst them, seropositivity with active TB was in 5 out of 27 patients (18.5%), and with post TB it was in 24 out of 49 patients (48.9% & p-value: 0.0070). The range of significant antibody levels found in post-tuberculosis patients was 27 mgA/l to 216 mgA/l. In a recently published study by Jha et al. from New Delhi, seropositivity rate was 38.9% in patients treated for TB [10]. In another study from Sierra Leone in Africa, the *Aspergillus* seropositivity is reported to be 20.8% as against 19.7% from Kenya in the post-tuberculosis patients [11,12]. In two studies from Indonesia, a total seropositivity of 43% was reported in patients treated for TB, 26.9% in bacteriologically confirmed TB patients, and 18.2% in those without bacteriological confirmation respectively [17,18].

In our study, in patients without a history of TB, the range of *Aspergillus fumigatus*-specific IgG antibody observed was 2.38 mgA/l to 105 mgA/l and significantly high antibody levels (>27 mgA/l) were found in 8 out of 42 patients (19.0%). The antibody levels were found to be statistically significantly high in the population with a history of tuberculosis in comparison with the non-tuberculosis group (p-value: 0.0355).

The limitation of the study is the lack of radiological evidence in the 19 cases. Hence, the prevalence of proven CPA (22 out of 128 clinically significant patients) in our study is 17.2%. If the chest radiography was included, the true prevalence in this population would have been considerably greater. Also, because of various socioeconomic reasons in this population, the cases may not report to the hospitals early enough for diagnosis.

The male to female ratio in the study was 1.6:1 with a male preponderance; the same was observed in the CPA cases as well. Similar findings were seen in studies done in France, Sierra Leone and Ghana [8,11,13]. However, the Kenya study showed a female preponderance [12].

In our study, patients in the age group of 21–50 years were predominant, and 31.9% of them had CPA [mean age 41.89 (±15.69)]. Similar findings were seen in Ghana (mean age 41.5 years) and Indonesian (mean age 44.4 years) studies [13,17]. However, in the Sierra Leone study, patients aged more than 45 years were having a higher prevalence of CPA [11]. In our study, 34.6% of the married people and 19.0% of the singles had CPA. However, the Sierra Leone study had 56.9% married patients in their study [11].

Almost half of the subjects in our study were working in the tea gardens or tea factories, while the other half of them were dependents of the tea garden workers who were students, unemployed, or employed in the same district doing various jobs (Mason, carpenter, plumber, manual labourers etc., or unemployed). Though there was no statistically significant difference in the occurrence of CPA between these two groups. In our study, CPA was more commonly seen in smokers (39.3%) than in non-smokers (29.5%) and did not have a statistical significance. CPA was predominant in non-smokers from another study from Kenya, in which 23.9% were smokers and 76.1% were non-smokers [12].

In our study, besides TB, other respiratory illnesses such as COPD and asthma were not found to be statistically significant as independent predictors of *Aspergillus* seropositivity. CPA was found in only 1 out of 13 (7.7%) asthma patients in our study. But the number of asthma patients in our study was also quite low. Studies elsewhere have shown a prevalence of 9.8% from Sierra Leone, 10.3% from Manchester and 3.9% from the Ghana study [11,13,19]. Out of 6 COPD patients, 3 had CPA in our study, which is 7.3% of the total CPA cases reported (3 out of 41 CPA cases). COPD was found to be a risk factor for CPA in 43.9% in a France study, whereas 2.4% and 5.8% were estimated in a study from Sierra Leone and Ghana, respectively [8,11,13].

In our study, 1.5% of the enrolled patients had chronic pulmonary aspergillosis with HIV, which is much lower than 6% as reported in a literature review from India by Denning et al. while in a Nigerian study, 10% of HIV-infected patients had CPA [4,20]. In another Mozambican study by Salzer et al. no CPA cases were found in HIV-positive TB patients [21].

Clinical symptoms most commonly associated with CPA were **haemoptysis and fatigue/tiredness** with a high statistical significance (p-values: **0.0086** and **0.0098**, respectively). This is consistent with case definition given by GAFFI [2]. In a similar study done by Jha et al. the most common symptoms in association with CPA were dyspnoea and haemoptysis (p-value: 0.066 and 0.011) [10]. In a study by Setianingram et al. cough, haemoptysis, and fatigue were significantly associated. Productive cough and dyspnoea were commonly present in patients with CPA in a Vietnamese study [22]. In African studies from Ghana, Sierra Leone, and Kenya, the symptoms most commonly found were cough and chest pain, followed by fatigue, weight loss, and night sweats [11–13].

The most significant radiological findings in CPA patients in our study were **single and multiple cavity** formations in the lungs followed by **fungal balls**. Nodules and consolidations were the most common findings in a study done by Jha et al [10]. However, the APICAL study states cavitation, pleural thickening, and infiltrates to be the most common radiological findings in CPA patients. While one study from Vietnam also states multiple cavities and pleural thickening as the common radiological findings [17,22]. Cavitation, para-cavitary fibrosis, and fungal ball were seen commonly in CPA patients in Ocansey et al.’s study from Ghana [13]. Infiltrates, cavitary changes, pleural effusion, pleural thickening, and fibrotic changes were significantly associated with CPA cases in Lakoh et al.’s study from Sierra Leone [11].

In our study, chronic cavitary aspergillosis (CCPA) was the most common type of CPA, followed by chronic fibrosing pulmonary aspergillosis and simple aspergilloma. Similarly, CCPA (88.9% & 3.9%) was the most common finding in Page et al. & Denning et al.’s studies [19,23].

Though the sample size is less in our study, we included cases from 3 tea garden hospitals and 2 referral hospitals. Therefore, the estimations of the prevalence of CPA in tea garden populations with chronic respiratory illness approximate its true prevalence.

## Conclusion

In this study, we provide the first evidence of *Aspergillus* seropositivity & CPA in the tea garden population of Assam. Looking at the high prevalence of CPA & *Aspergillus* seropositivity in the total TB (38.1%) and post-TB (48.9%) patients with radiological features consistent with CPA, we would like to suggest testing for *Aspergillus* antibody in patients who come with respiratory symptoms after completion of tuberculosis treatment in order to diagnose the cases early and treat them subsequently. This will help to decrease the morbidity & mortality due to this neglected and underdiagnosed disease in tea garden hospitals as well as other TB hospitals.

## Supporting information

S1 Annexure Performa used for collection of patient details. (DOCX)

S2 Annexure Consent form used for obtaining consent for performing the test in the patient. (DOCX)

S1 Fig HRCT image of a 26-year-old male with chronic cough in the last 3 months showing fungal ball in right lung lobe. (TIF)

S2 Fig 47-year-old TB defaulter-Fungal ball with lung volume loss with a cavity showing fungal ball (red arrow) in left lung (CFPA). (TIF)

S3 Fig 25-year-old female presented with chronic cough and chest pain in the last 3–4 months, HRCT showing intraluminal material (Orange arrow) in left lung, CCPA. (TIF)

S4 Fig 25-year-old female presented with chronic cough and chest pain for 3–4 months. Sagittal view with multiple bronchiectasis and 2 large cavities with intraluminal materials (green arrows), CCPA. (TIF)

S5 Fig 56-year-old post-TB patient’s right lung showing consolidation with bronchiectasis-Chronic Fibrosing Pulmonary Aspergillosis. (TIF)

S6 Fig 30-year-old male from Dibrugarh, HIV positive, TB positive presented with fever, cough and breathlessness, Fungal ball with pericavitary fibrosis (orange arrow) and pleural thickening (yellow arrow) in left lung lobe in axial view. (TIF)

S7 Fig ImmunoCAP Report Page1 showing Calibrator curve for the samples processed. (TIF)

S8 Fig ImmunoCAP Report Page2 showing Calibrator values obtained and IgG concentration values of 12 samples used in the first run. (TIF)
