# Supplementary material for: Chronic pulmonary aspergillosis in tea population of Assam
Source: PLoS Negl Trop Dis. 2025 Jan 8;19(1):e0012756. doi: 10.1371/journal.pntd.0012756 (PMC11709265; doi:10.1371/journal.pntd.0012756)
Supplement: S1 Annexure — (DOCX) [file pntd.0012756.s001.docx]

**ANNEXURE-I**

**PERFORMA**

**General Information:**

- Code :
- Age :
- Gender :
- Occupation : (tea plucker or working in a tea factory)
- Tea Garden name :

**Personal Information :**

- Marital status :
- H/O Smoking :
- H/O Alcohol consumption :

**Morbidity Status :**

- Pulmonary TB – Present or absent :
- If present smear positive or negative :

1. Date of starting treatment :
2. Duration of treatment :
3. Treatment completed or cured :

- Co-morbidities :

1. HIV :
2. Diabetes mellitus :
3. COPD / Asthma :
4. Others :

**Symptoms:** (duration to be mentioned)

Weight loss :

Fever :

Breathlessness :

Chronic cough :

Hemoptysis :

Fatigue/ tiredness :

**Clinical Examination:**

- Lymph node enlargement :
- Others:

**Radiological Findings:**

Nodules in chest x-ray :

Cavitations :

Para-cavitary fibrosis :

Fibro-nodule :

Infiltrates :

Fungal ball :

Consolidation :

Pleural thickening :

Others if any :
