# Supplementary material for: Chronic pulmonary aspergillosis in tea population of Assam
Source: PLoS Negl Trop Dis. 2025 Jan 8;19(1):e0012756. doi: 10.1371/journal.pntd.0012756 (PMC11709265; doi:10.1371/journal.pntd.0012756)
Supplement: S2 Annexure — (DOCX) [file pntd.0012756.s002.docx]

**ANNEXURE-II**

Code

INFORMED CONSENT FORM

Informed consent form for patients with respiratory symptoms from the tea garden hospitals and the tea garden community within 15km radius of Assam Medical College, from whom serum samples, HRCT or CXR will be collected after being requested to participate in the research titled **“Chronic Pulmonary Aspergillosis in tea population of Assam”**

**Name of Investigators : DR. AISHWARYA K.** PGT, Department of Microbiology, AMCH

The informed consent form has two parts:

- PART I: Information sheet (to share information of the study with you)
- PART II: Certificate of Consent (for signatures if you choose to participate)

**PART I: Information sheet**

I am **Dr. K. Aishwarya,** a postgraduate trainee in the department of microbiology of Assam Medical College and Hospital. I am doing a study on “**Chronic Pulmonary Aspergillosis in tea population of Assam**” in patients having chronic respiratory symptoms. I am going to give you information and invite you to participate in this research. This consent form may contain words that you do not understand. Please ask me to stop as we go through the information and I will take time to explain.

**Purpose of the Study:** To evaluate the prevalence of *Aspergillus* antibody in the people of tea garden community of Dibrugarh, Assam by collecting their serum sample to confirm the presence of the same in suspected patients with chronic respiratory symptoms.

**Description of the Process:** During the study when you will be referred to the tea garden hospital, I will be asking you questions in the proforma and will do the general examination. Serum samples will be collected and chest x-ray and HRCT will be done according to the standardized procedure.

Your participation is entirely voluntary. It is your choice whether to participate or not. You do not have to answer any question if you do not wish to.

There will be no direct benefit to you or any reimbursements provided for participating in the study.

**RISKS**

**Confidentiality:** We will not be sharing information about you to anyone outside of the research team. Any information about you will have a number on it instead of your name. Only the researchers will know what your number is. All information will be kept private and confidential and nothing will be attributed to you by name. The knowledge that we get from this research will be shared with you. We will publish the results so that other interested people may benefit from the research.

You do not have to take part in the research if you do not wish to do so. You may stop participating in the study anytime you wish to.

**CONTACT PERSON**

This proposal has been reviewed and approved by Assam Medical College Institutional Review Board (IRB) which is a committee whose task it is to make sure that research participants are protected from harm.

If you wish to contact IRB for any queries, phone number is 0373-2300080.

If you wish to ask me any questions later, my mobile number is 9962788801.

**PART II: CERTIFICATE OF CONSENT**

I have read the foregoing information, or it has been read to me. I have had the opportunity to ask questions about it and any questions I have asked have been answered to my satisfaction. I consent voluntarily to be a participant in this study.

Name of the Participant :

Signature of the Participant :

Date:

If illiterate, Thumb Print of Participant:
