# Supplementary figures and images for: Chronic pulmonary aspergillosis in tea population of Assam
Source: PLoS Negl Trop Dis. 2025 Jan 8;19(1):e0012756. doi: 10.1371/journal.pntd.0012756 (PMC11709265; doi:10.1371/journal.pntd.0012756)

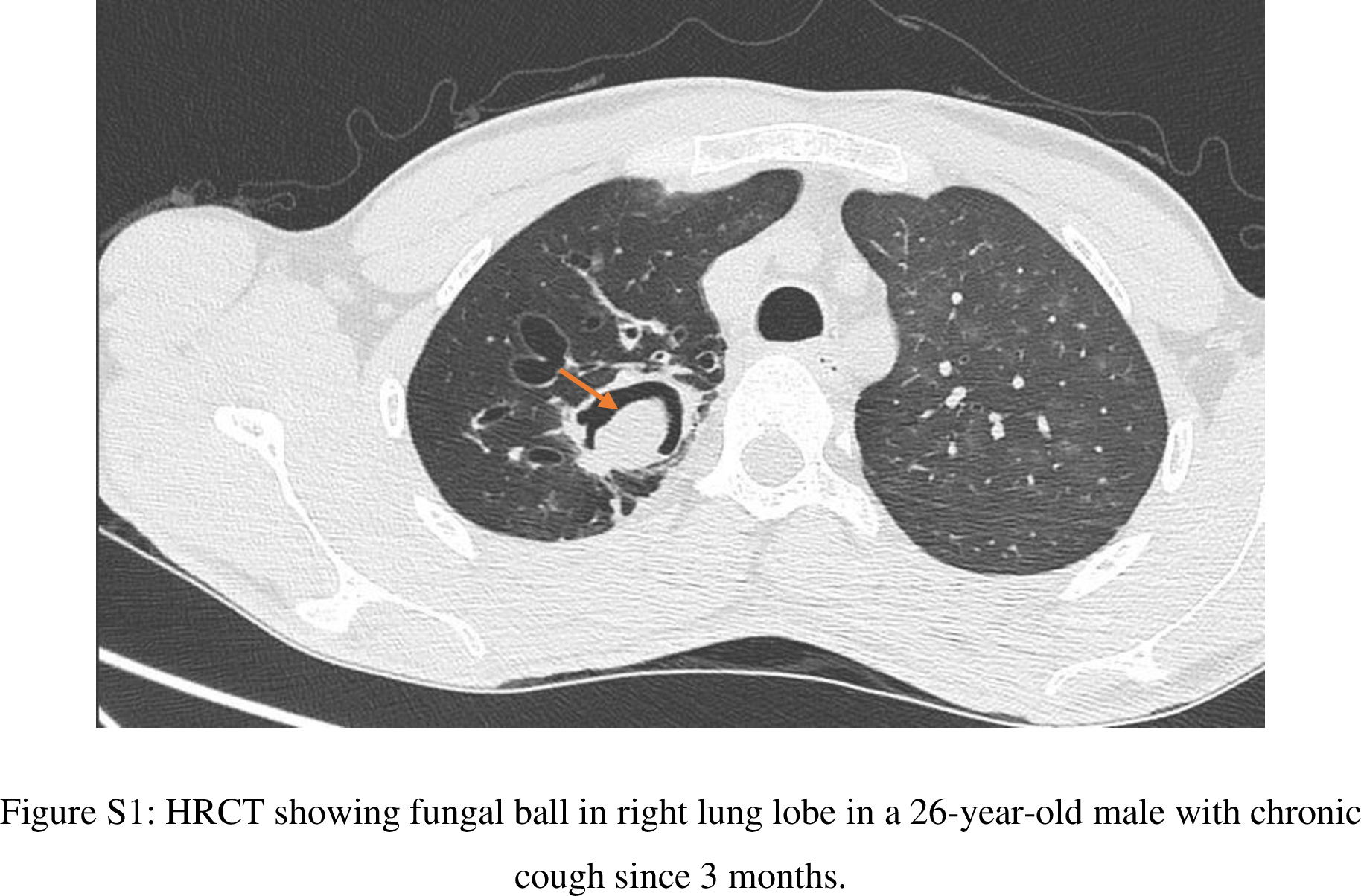

Supplement: S1 Fig — (TIF) [file pntd.0012756.s003.tif]

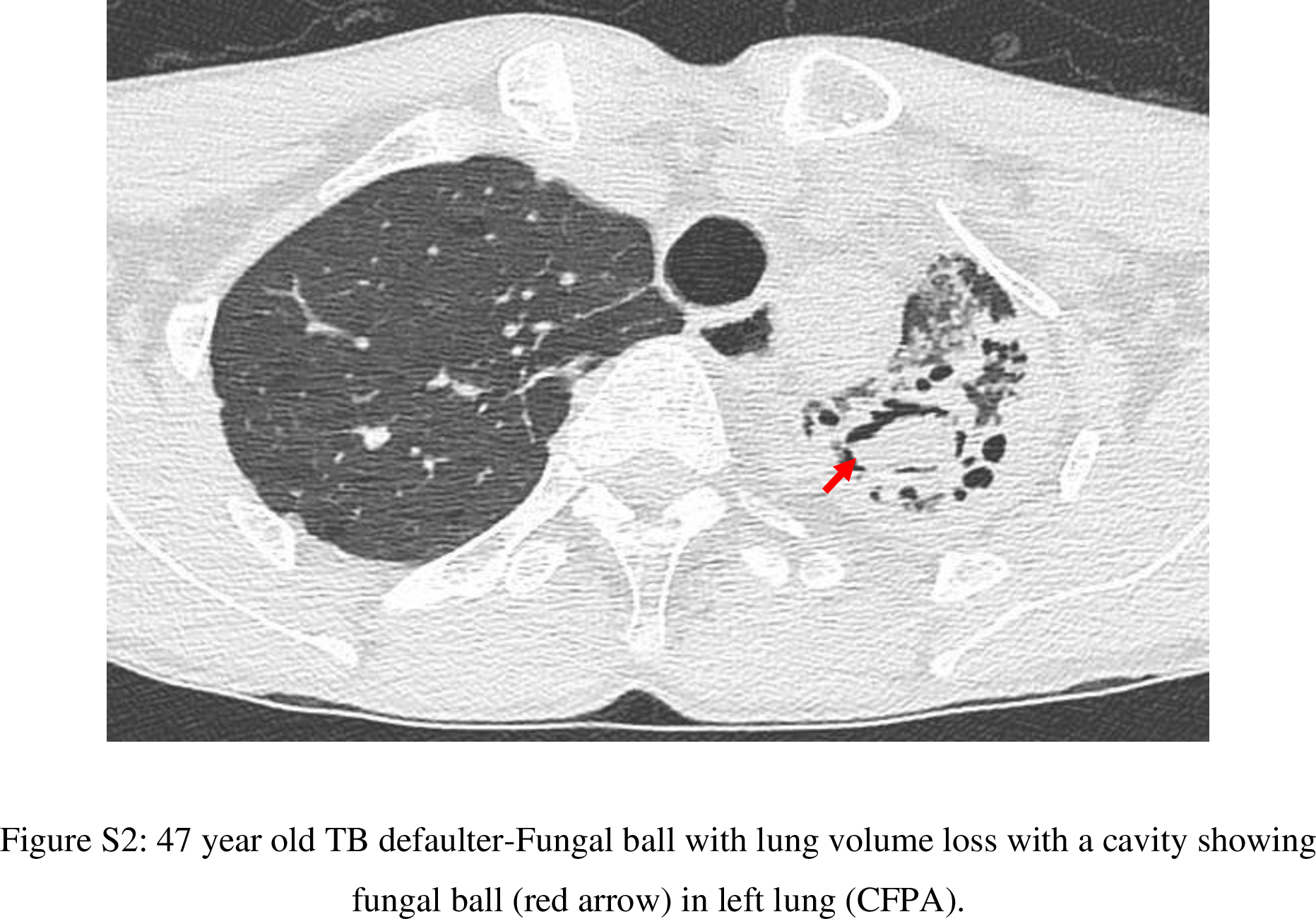

Supplement: S2 Fig — (TIF) [file pntd.0012756.s004.tif]

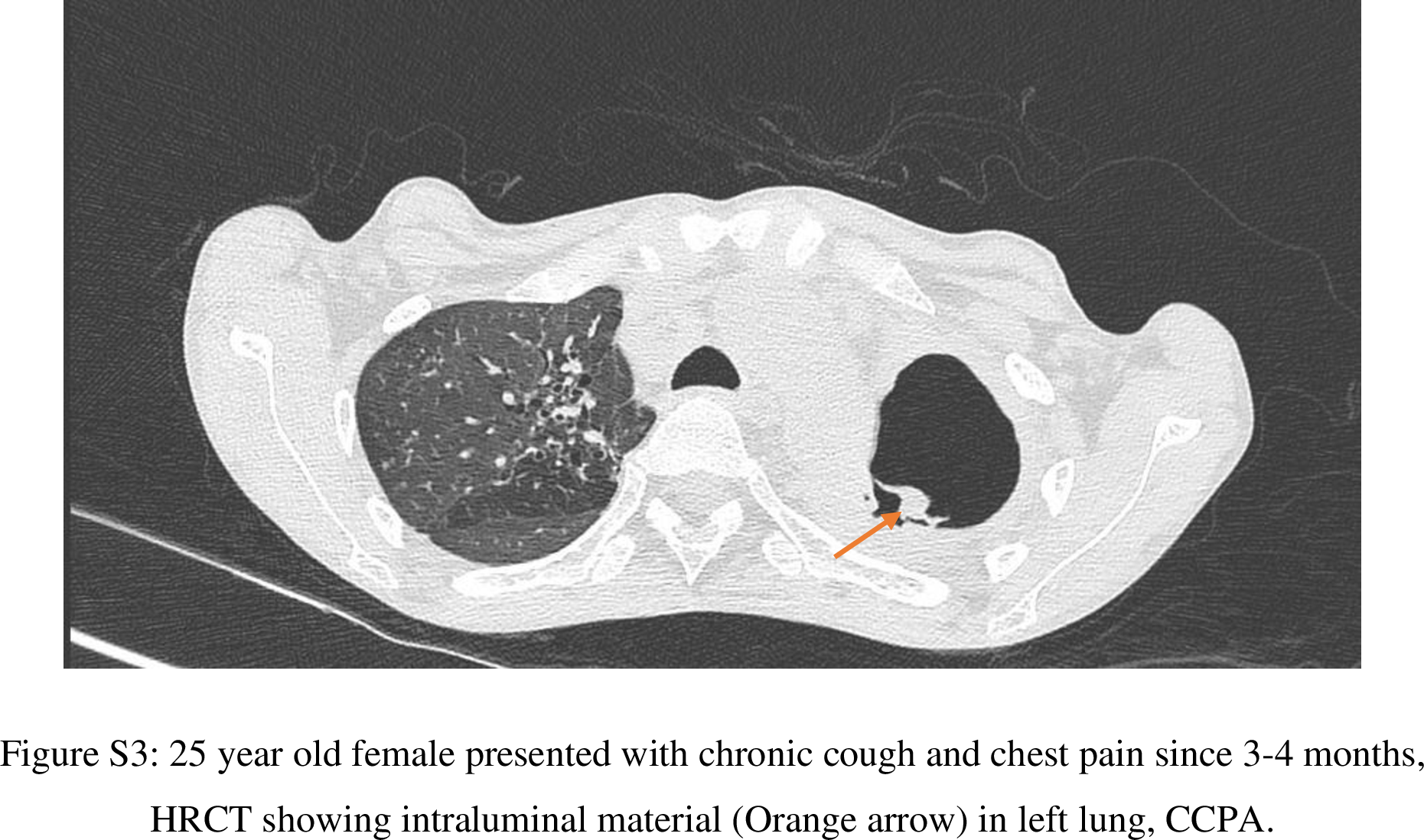

Supplement: S3 Fig — (TIF) [file pntd.0012756.s005.tif]

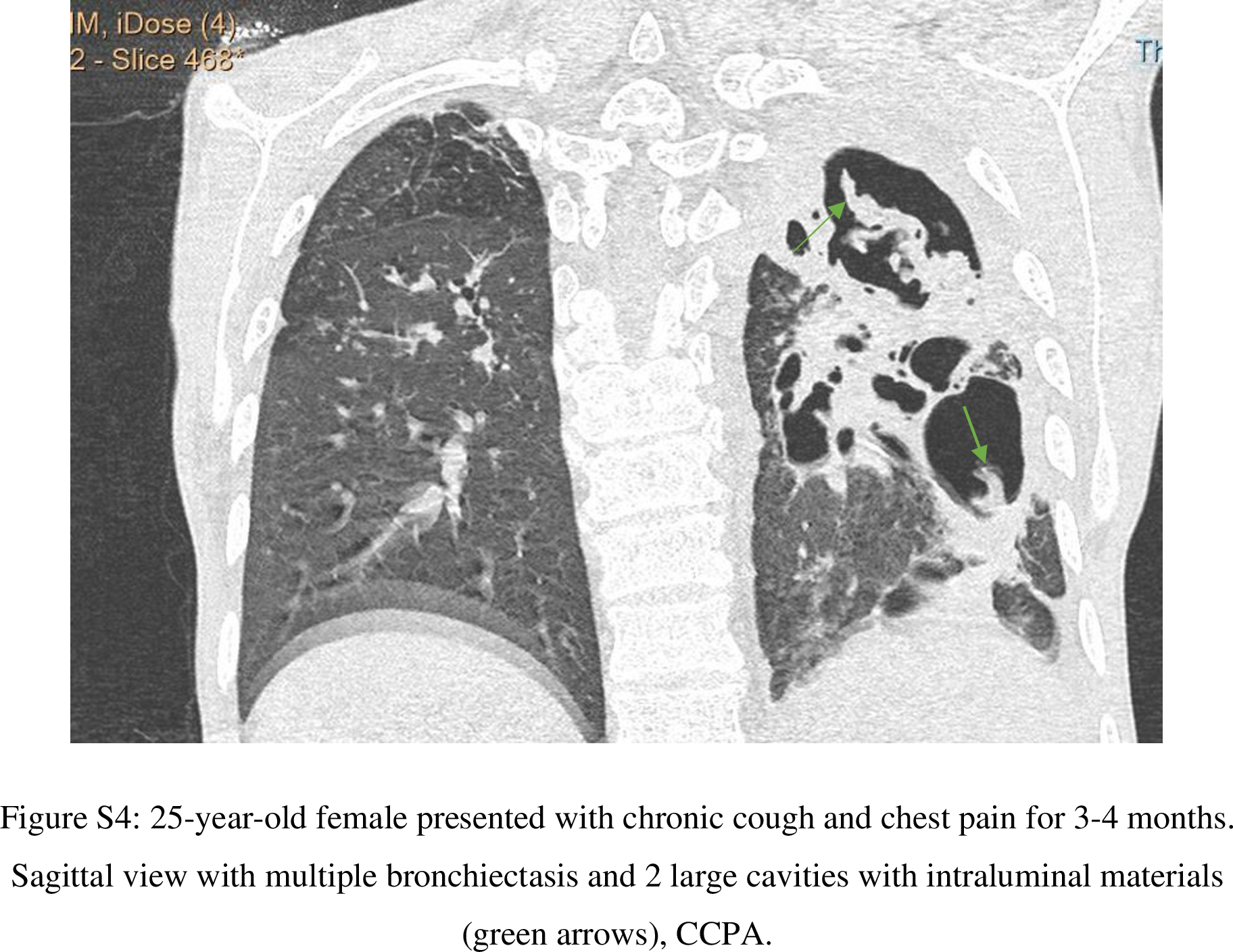

Supplement: S4 Fig — (TIF) [file pntd.0012756.s006.tif]

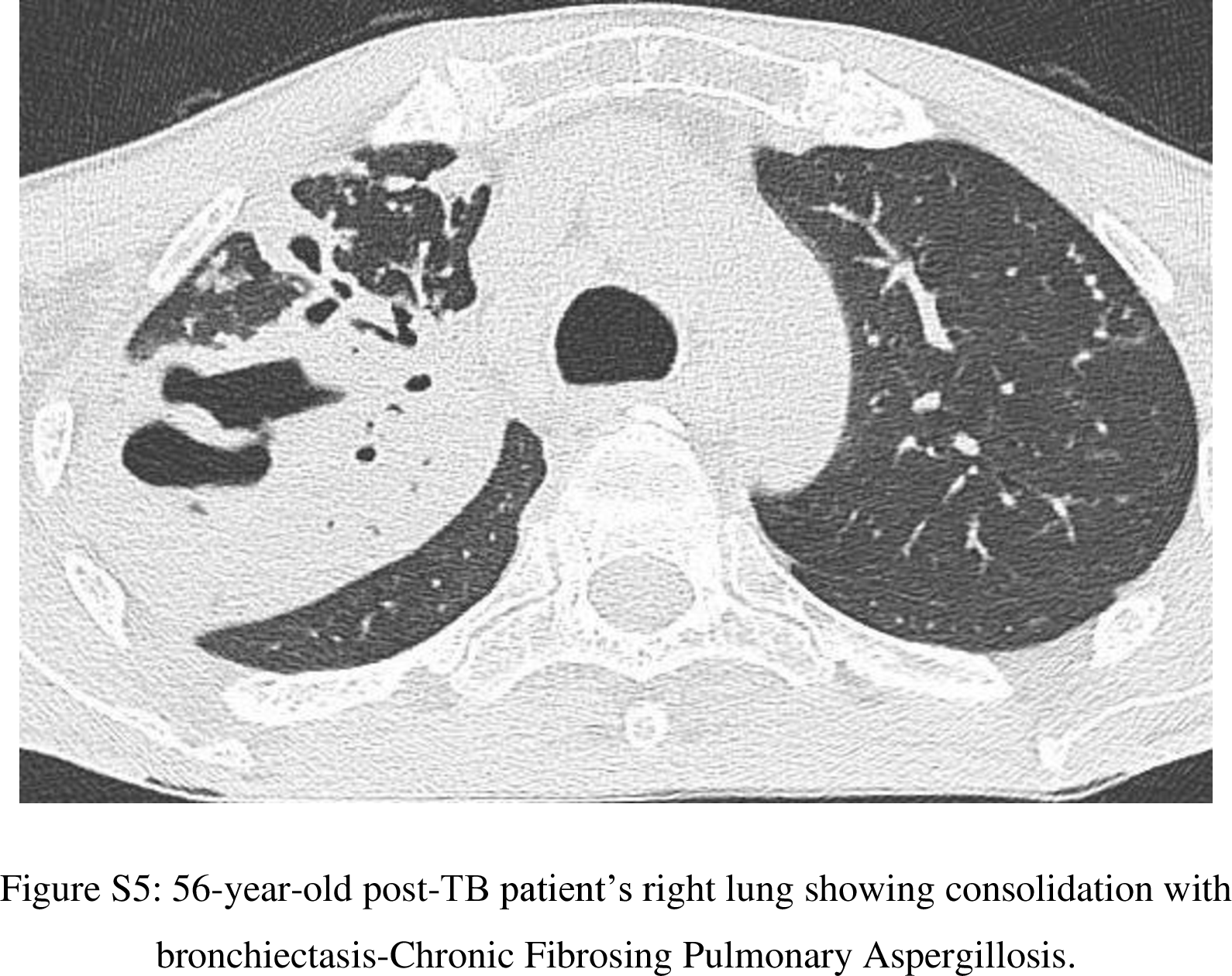

Supplement: S5 Fig — (TIF) [file pntd.0012756.s007.tif]

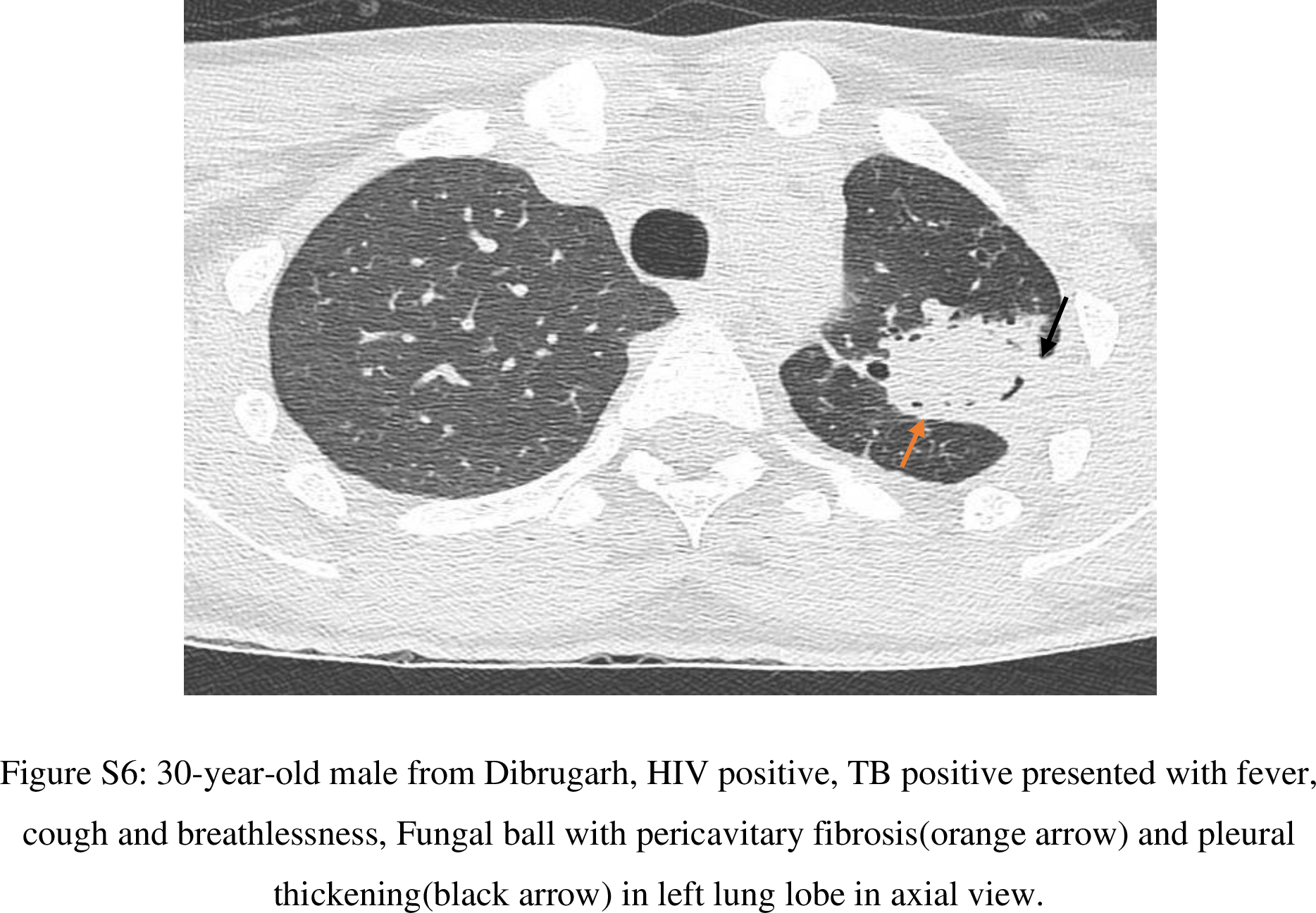

Supplement: S6 Fig — (TIF) [file pntd.0012756.s008.tif]

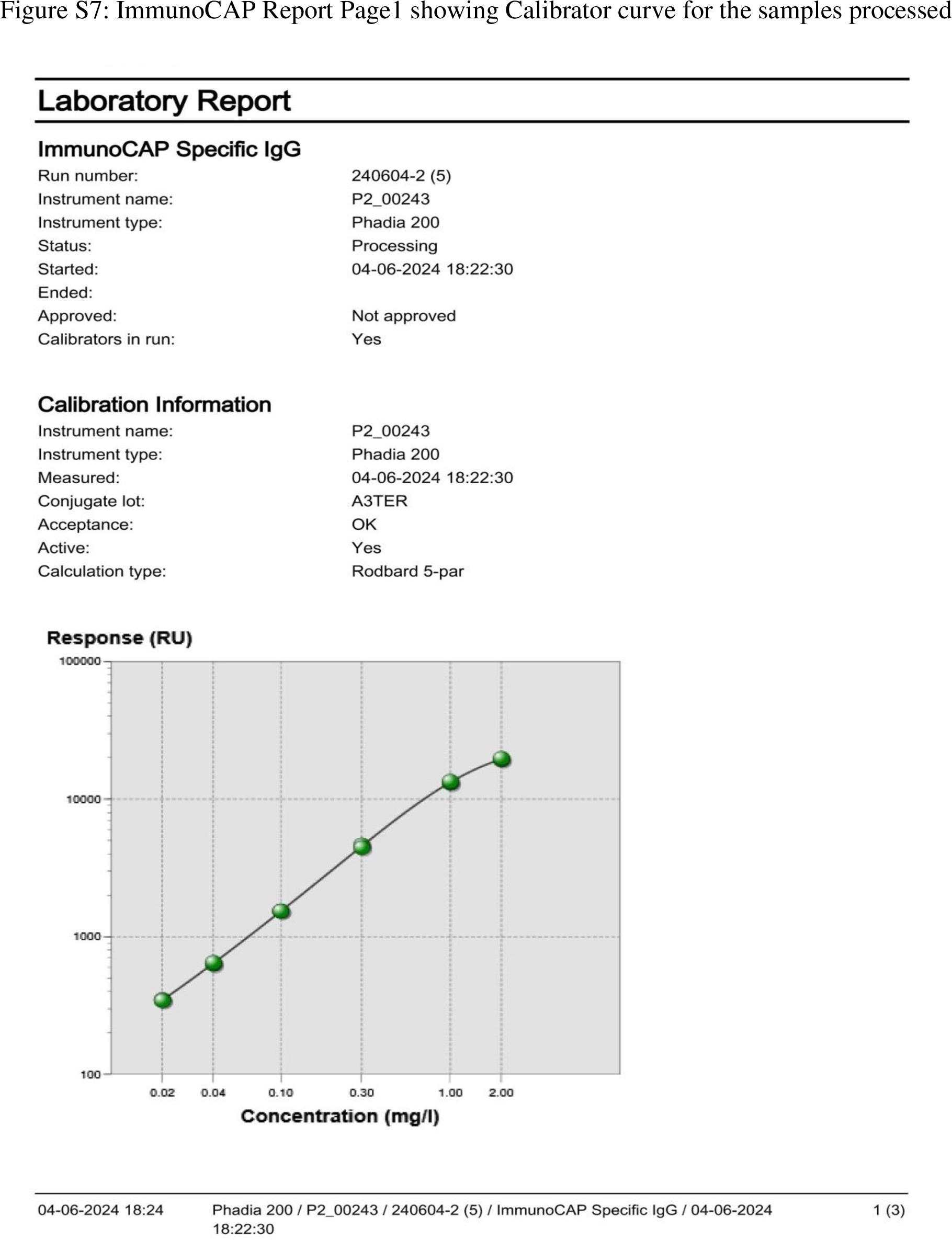

Supplement: S7 Fig — (TIF) [file pntd.0012756.s009.tif]

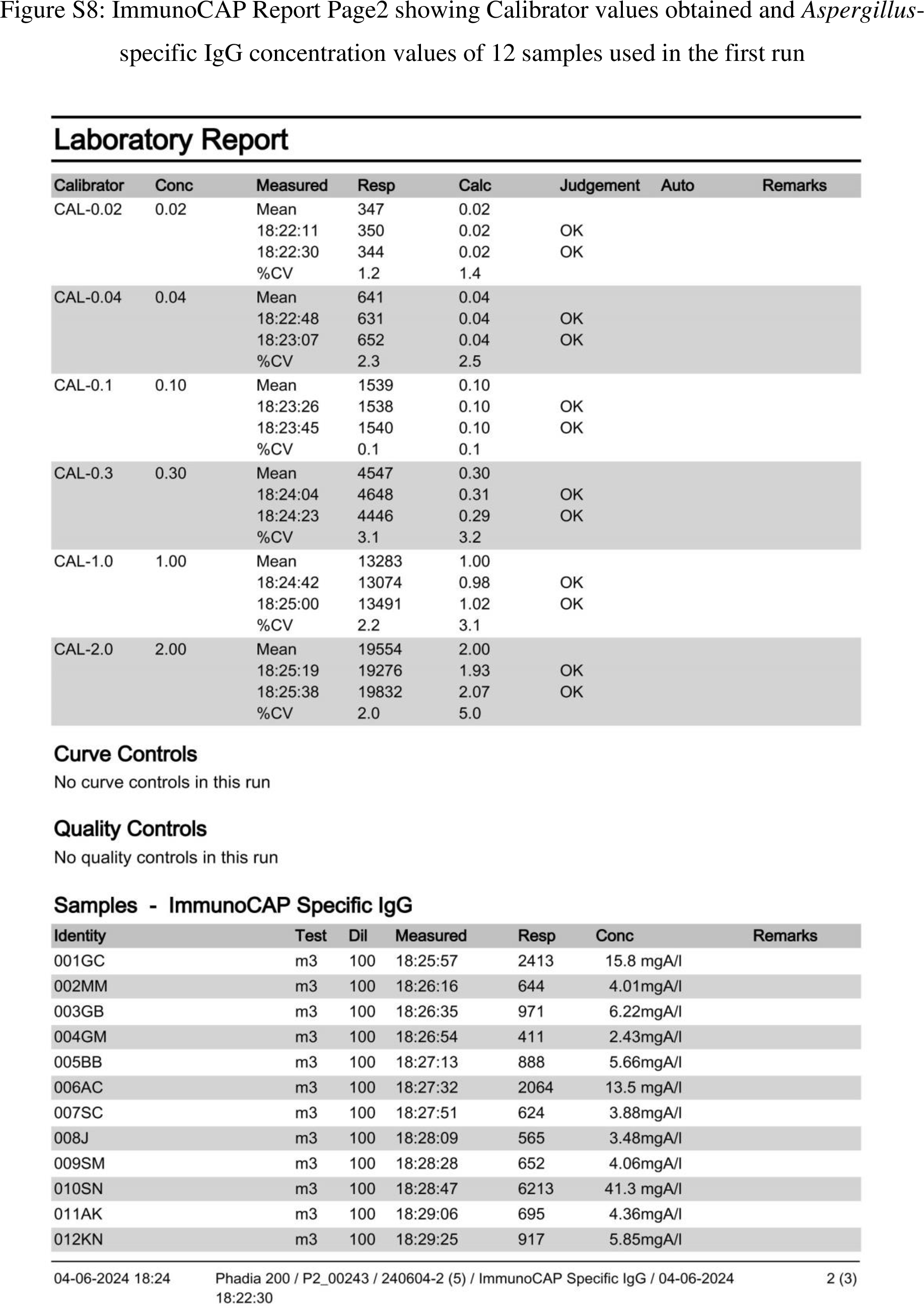

Supplement: S8 Fig — (TIF) [file pntd.0012756.s010.tif]
